# Time course of organ and hematological response to complement blockage in transplant-associated thrombotic microangiopathy after allogeneic hematopoietic stem cell transplantation

**DOI:** 10.3389/fmed.2025.1551066

**Published:** 2025-03-27

**Authors:** Tatiana A. Rudakova, Julia Yu Vlasova, Olesya V. Paina, Olga A. Slesarchuk, Marina A. Gorodnova, Tatiana S. Schegoleva, Oleg V. Goloshchapov, Tatiana A. Bykova, Elena V. Morozova, Lyudmila S. Zubarovskaya, Ivan S. Moiseev, Alexander D. Kulagin

**Affiliations:** RM Gorbacheva Research Institute, Pavlov University, Saint Petersburg, Russia

**Keywords:** hematopoietic cell transplantation, early complications, transplantation-associated thrombotic microangiopathy, complement inhibition, eculizumab

## Abstract

**Background:**

Hematopoietic stem cell transplantation (HSCT) offers a potential cure for various hematologic malignancies and non-malignant disorders but is often accompanied by severe complications, one of the most challenging being transplant-associated thrombotic microangiopathy (TA-TMA). Eculizumab, a complement inhibitor, has emerged as an effective therapeutic option for TA-TMA.

**Methods:**

This single-center retrospective study was conducted at Pavlov University, St. Petersburg, to evaluate the efficacy of eculizumab in 14 adult and pediatric patients who developed high-risk TA-TMA following HSCT between 2015 and 2023. Treatment response was assessed by monitoring organ functions, blood counts, transfusion requirements, the presence of schistocytes in peripheral blood, and increased serum lactate dehydrogenase (LDH). The primary endpoint was overall survival at 100 days from eculizumab administration. Secondary endpoints included the cumulative incidence of a 25% decrease in serum lactate dehydrogenase levels and to the limit of the normal range for age from the date of the initiation of eculizumab, the cumulative incidence of a 50% increase in platelet count or stable platelet levels ≥ 20×10^9^/l was 74% (95% CI, 32–92) with median time 21 days (range: 1–104), cumulative incidence of platelet level ≥ 50×10^9^/l, and 1 year from the date of the initiation of eculizumab.

**Results:**

Overall survival at 100 days was 57% (95%CI, 36–90). The cumulative incidence of LDH decreased by 25% was 89% (95% CI, 26–99) with a median time of 11 days (range: 2–27). Cumulative incidence of LDH ≤ 1.5 upper reference limits (URLs) after eculizumab therapy was 73% (95% CI, 34–91) with a median time of 22 days (range: 2–170). The cumulative incidence of a 50% increase in platelet level or stable platelet level ≥ 20×10^9^/l was 74% (95% CI, 32–92) with a median time of 21 days (range: 1–104). The cumulative incidence of platelet level ≥ 50×109/l was 56% (95% CI, 22–80) with a median time of platelet increase of 75 days (range: 5–384). Complete response was documented in 57% of the group.

**Discussion:**

In summary, eculizumab is a well-tolerated promising therapeutic intervention for TA-TMA, but more studies are needed to establish its timing and dosage regimen in TA-TMA.

## Introduction

Allogeneic hematopoietic stem cell transplantation (allo-HSCT) is an effective therapeutic strategy for patients with various hematologic malignancies and non-malignant disorders. Despite its curative potential, allo-HSCT is associated with severe complications, e.g., microvascular complications, the pathogenesis of which involves damage and dysfunction of the endothelium. Among them, transplant-associated thrombotic microangiopathy (TA-TMA) is considered one of the most challenging, life-threatening conditions. It manifests with thrombosis, hemolysis, and multiorgan dysfunction, affecting 20 to 30% of allo-HSCT recipients (1).

The pathophysiology of TA-TMA involves a complex interplay between endothelial damage, complement activation, and immune dysregulation. The endothelium is a highly active cell community that plays a significant role in the regulation of vascular tone, coagulation, and inflammation. The membrane attack complex attracts and activates platelets, causing the release of von Willebrand factor from endothelial cells. Activation of complement components C3a and C5a induces platelet activation and aggregation, thus triggering the cytokine release. This process of local complement activation may increase the risk of local blood clotting (2–4).

A “Two-hit” model was proposed to describe the pathogenesis of TA-TMA. Normal endothelium transforms into procoagulant endothelium as a result of various risk factors present during the preconditioning and early post-HSCT period. Factors that initiate endothelial injury affect this procoagulant endothelium, resulting in thrombotic microangiopathy (5). Jodele et al. suggested that the onset of TA-TMA requires at least two factors: a genetically susceptible host and environmental stressors; both factors might differ in their strength and contribution to the development of TA-TMA (6). Several risk factors may be associated with the development of TA-TMA, most notably drugs that are used in condition regimens and graft-versus-host disease (GvHD) prophylaxis, infections, and other post-transplant complications (7). Such agents as busulfan, fludarabine, cisplatin, and radiation, as well as calcineurin inhibitors and mammalian target of rapamycin (mTOR) inhibitors, are known to be associated with increased risk of TA-TMA (8). Meta-analysis performed by Van Benschoten et al. named acute GVHD as the most common risk factor (9). Additionally, bacterial and viral infections are often considered potential triggers for TA-TMA (8).

The clinical course of TA-TMA is characterized by microangiopathic hemolytic anemia with persistent schistocytosis, thrombocytopenia, and microvascular thrombosis leading to ischemic injuries in the various organs. An expert panel of the American Society for Transplantation and Cellular Therapy, Center for International Bone Marrow Transplant Research, Asia-Pacific Blood and Marrow Transplantation, and European Society for Blood and Marrow Transplantation has agreed on using the modified Jodele criteria with additional definitions of anemia and thrombocytopenia (7, 10).

There is no uniformly agreed treatment protocol for TA-TMA, though a number of care strategies have been introduced. Such measures as discontinuation of calcineurin inhibitors, therapeutic plasma exchange, and pharmacological options such as rituximab, defibrotide, and daclizumab have been described, but due to the variability among patient groups and the absence of consistently applied diagnostic criteria for TA-TMA, it is difficult to compare different strategies of TA-TMA management (11).

The pivotal role of complement activation in the pathophysiology of TA-TMA has stimulated the search for the therapeutic potential of complement inhibitors in the posttransplant setting. Eculizumab, being a terminal complement path inhibitor, has shown promise in treating TA-TMA. Most studies in adult cohorts adopted the standard regimen of eculizumab administration for atypical hemolytic uremic syndrome, which involves an initial induction therapy of 900 mg weekly for 4 weeks, followed by maintenance therapy of 1,200 mg every 2 weeks once hematological signs of TA-TMA resolve, while body weight adjustment was applied for the pediatric patients. Muzino et al. published a model-based optimal loading dosing protocol for bleeding and non-bleeding patients, identifying that bleeding patients need prolonged therapy courses (12). Studies assessing eculizumab efficacy in TA-TMA have reported a hematological response rate of 70 to 93% and survival rates between 60 and 67%, indicating variability in outcomes across different research (13).

Our study aimed to contribute to the efficacy of this therapeutic approach of TA-TMA treatment with eculizumab by presenting a single-center real clinical experience.

## Materials and methods

We retrospectively reviewed records of consecutive patients who presented with a diagnosis of TA-TMA and were treated with eculizumab between 2015 and 2023 at our institution.

Patients’ blood count, presence of schistocytes, LDH levels, transfusion requirements, proteinuria, blood pressure, and renal function were monitored. Anemia and thrombocytopenia were graded according to Common Terminology Criteria for Adverse Events (CTCAEs), version 5. All patients had PCR screening for gut colonization and blood cytomegalovirus, human herpes virus types 1, 2, and 6, two times weekly.

The primary endpoint was OS at 100 days from eculizumab administration. The secondary endpoints were cumulative incidence of 1) LDH decrease by 25% and ≤ 1.5 upper limit of normal (ULN), 2) a 50% increase in platelet levels or a stable platelet level of ≥ 20×10^9^/L, 3) an increase in platelet levels ≥ 50×10^9^/L, 4) an increase in hemoglobin levels by at least 1 g/dL without red blood cell transfusions after the initiation of eculizumab therapy, all assessed on 100 days and 1 year from the date of the initiation of eculizumab.

TA-TMA was diagnosed using criteria by Cho et al. (4) before 2022 and TMA Harmonization Panel Consensus Recommended Diagnostic Criteria (2022) after 2022, depending on the year of allo-HSCT (Supplement) (4, 7). Due to limited availability, sC5b-9 monitoring was not performed. No patients underwent biopsy for the diagnosis of TA-TMA.

Kidney Disease: Improving Global Outcomes (KDIGO) criteria were used to define and stage acute kidney injury (AKI).

Patients were stratified according to the following risk groups: standard-risk TA-TMA (peak LDH <2 times ULN, spot rUPCR <1 mg/mg, KDIGO stage I AKI) and high-risk TA-TMA (peak LDH >2 times ULN, spot random urine protein/creatinine ratio (rUPCR) ≥1 mg/mg, any organ dysfunction developing in the setting of TMA except KDIGO stage I AKI, concurrent acute GVHD grades II–IV, concurrent systemic bacterial or viral infection) (4).

### Treatment with eculizumab

Due to limited access to the medication, treatment with eculizumab was indicated only to high-risk TMA patients, and the dosage regimen was 600 mg weekly. TA-TMA and multiple organ failure response were evaluated after the last eculizumab dose, and survival was assessed at 100 days from TA-TMA diagnosis and 1 year after allo-HSCT.

Response to treatment was defined by the improvement of the clinical and/or laboratory diagnostic parameters. Complete response (CR) to eculizumab was defined as resolution of multiple organ failure, independency of red cell and platelet transfusions, and resolution of at least one marker of microangiopathy (normalization of serum LDH levels or absence schistocytes), and responding patients who did not achieve CR were categorized as partial response (PR). Non-responders were considered to be those patients who remained dependent on transfusions, did not recover vital organ function after the last eculizumab dose, or died with active TA-TMA.

### Statistics

Patient and transplant characteristics were evaluated by means of descriptive statistical software. All patients were followed longitudinally until at least 100 days after HSCT or until TA-TMA resolution or death. Cumulative incidence rates and their 95% confidence intervals were estimated for LDH and platelet levels from the date of the first dose of eculizumab with death as a concurrent event. Kaplan–Meier analysis was used to estimate OS and time to response. All statistical procedures were performed with R free software package v. 4.3.0.

## Results

### Baseline characteristics

We retrospectively analyzed a diverse cohort of 14 patients, including 8 adults and 6 pediatric patients with a median age of 27 years (1–62) (Table 1). The sources of stem cells were variable, using bone marrow transplants in half of the clinical cases. The conditioning regimens were predominantly oral busulfan-based at a total dose of 8–16 mg/kg. GVHD prophylaxis included was mostly cyclophosphamide-based and included calcineurin inhibitors in 86%. The median pre-HSCT creatinine level for children was 0.047 (range: 0.025–0.169) mmol/l, while for adults it was 0.075 (range: 0.05–0.1) mmol/l. Baseline glomerular filtration rate (GFR) before allo-HSCT in children was 156 (range: 80–197) ml/min, whereas in adults, it was 99 (range: 74–156) ml/min/1.73 m2.

**Table 1:** Characteristics of patients with TA-TMA.

Variable	Total subjects, *n* (%)
Male	9 (64)
Female	5 (36)
Age at HSCT, mediane (range), years	**27 (1–62)**
Children	6 (43)
Adults	8 (57)
Diagnostic group	
Acute lymphoblastic leukemia (ALL)	3 (21.4)
Acute myeloid leukemia (AML)	3 (21.4)
Myelodysplastic syndrome (MDS)	2 (14.3)
Primary myelofibrosis (PMF)	1 (7.14)
Hodgkin’s lymphoma (HL)	1 (7.14)
Shwachman-Diamond syndrome (SDS)	1 (7.14)
Inherited dyskeratosis	2 (14.3)
Osteopetrosis	1 (7.14)
Stem cell donor type	
Related (MRD, haplo)	8 (57)
Unrelated (MUD, MMUD)	6 (43)
HLA match	
Fully matched (MRD, MUD)	5 (36)
Mismatched (MMUD, haplo)	9 (64)
Stem cell source	
Bone marrow (BM)	6 (43)
Peripheral blood (PBSC)	7 (50)
BM + umbilical blood	1 (7)
Conditioning regimen type	
Myeloablative	8 (57)
Reduced intensity	6 (43)
GVHD prophylaxis	
Cyclophosphamide-based	9 (64)
Antithymocyte globulin based	2 (14)
Other	3 (22)
Calcineurin inhibitors	
Yes	12 (86)
Other	2 (14)

MRD, matched related donor; haplo, haploidentical donor; MUD, matched unrelated donor; MMUD, mismatched unrelated donor.

### Clinical presentation of TA-TMA onset

TA-TMA was diagnosed during the first 100 days after transplantation in 10 of 14 (92%) subjects with a median time from allo-HSCT to the TA-TMA diagnosis of 34 days (range: 13–62). Four patients exhibited TA-TMA after day 100, and all of them underwent prolonged therapy for GVHD.

All 14 patients had coincident conditions at the time of TA-TMA diagnosis: active infection (11 of 14, of them 5 patients had sepsis), acute GVHD 3–4 grade (2 of 14), acute and overlap GVHD complicated by both viral reactivation and bacterial infection (4 of 14), severe chronic GVHD complicated by bacterial infection (1 of 14), and one patient had signs of cytokine release syndrome at the time of TA-TMA; thus, all patients included had high-risk criteria. Other clinical characteristics are presented in Table 2.

**Table 2:** Clinical presentation of TA-TMA in patients.

Characteristics	Number of patients <18 years old	Number of patients > 18 years old	Total, *n* (%)
Anemia	6	6	12 (86)
Thrombocytopenia	6	8	14 (100)
Schistocytes, %	10 (2–54)	13 (2–41)	14 (100)
LDH, E/l: median (range)	1 patient 2 years old: 665 4 pts.: 6–9 years old 1858 (760–2,611) 1 patient 14 years old: 1993	611 (452–3,206)	14 (100)
Creatinine, mmol/l: median (range)	0.126 (0.04–0.432)	0.170 (0.07–0.353)	14 (100)
Proteinuria	4	8	12 (86)
Hypertension	2	3	5 (36)
Pulmonary bleeding	1	0	1 (7)
Gastrointestinal bleeding	1	2	3 (21)
CNS involvement	2	4	6 (42)
Admission to intensive care unit	3	2	5 (36)
Renal replacement	1	3	4 (29)

The median maximal creatinine level during TA-TMA was 0.126 (range: 0.038–0.432) mmol/l for children and 0.170 (range: 0.07–0.353) for adults. The median lowest GFR for children was 100 (range: 19–174) ml/min, and for adults, − 35 (range: 17–131) ml/min.

Five patients had severe TA-TMA-associated arterial hypertension. Acute renal injury and proteinuria during the TA-TMA course were revealed in 12 patients, with 3 cases of proteinuria on baseline urinalyses. Renal replacement therapy was needed in 4 of 14 patients.

CNS involvement before eculizumab infusion was documented in 6 of 14 cases (43%); of them, 1 patient had seizures, 1 patient had generalized tremor, and 4 patients suffered from reversible encephalopathy syndrome.

Five of 14 patients (36%) have been treated at the intensive care department, and all of them suffered from multiple organ failure.

### Treatment of TA-TMA

All patients with calcineurin inhibitor-based GVHD prophylaxis either discontinued intake of calcineurin inhibitors or were switched to sirolimus depending on GVHD activity as the first management procedure after TA-TMA diagnosis. All patients received infusions of intravenous human immunoglobulins 0.4–1 mg/kg weekly. Two pediatric patients underwent plasmapheresis prior to eculizumab administration.

Taking into account the infectious status of the patients at the time of TA-TMA onset, all of them received antibiotic therapy according to local protocol for infectious complications. No vaccination against encapsulated bacteria was performed.

The median cumulative dose of eculizumab was 1,200 mg (range: 600–1800) for children and 1,200 mg (range: 600–3,600) for adults. Eculizumab was successfully discontinued in all survivors after TA-TMA resolution.

### Response to eculizumab

**Figure 1:**
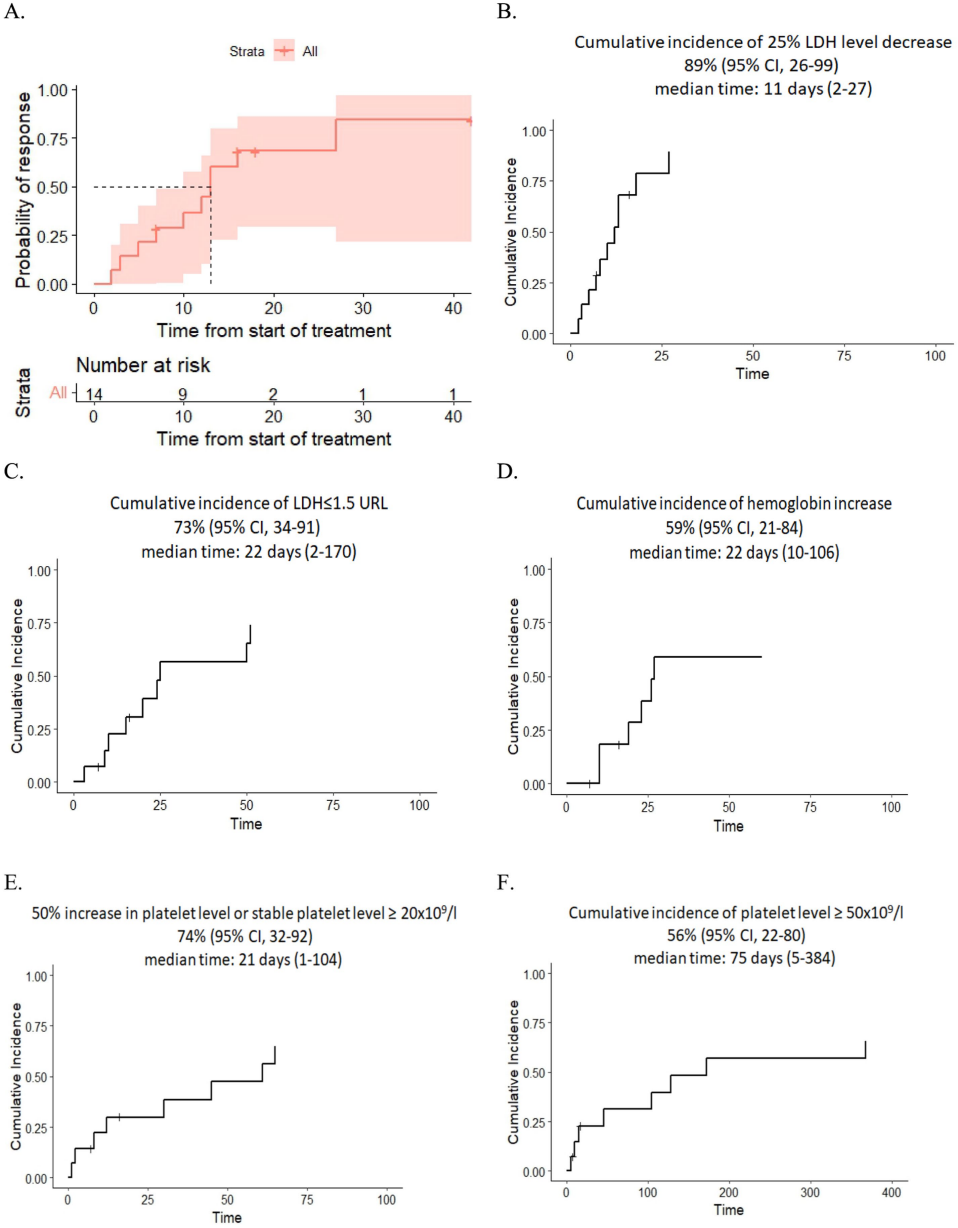
**(A)** Kaplan–Meier plot for time to response. **(B,C)** Dynamics of LDH level after eculizumab therapy. **(D)** Dynamics of hemoglobin level after eculizumab therapy. **(E,F)** Dynamics of platelet level after eculizumab therapy.

Resolution of multiple organ failure, independency of red cell and platelet transfusions, and resolution of at least one marker of microangiopathy (normalization of serum LDH levels or absence of schistocytes), i.e., CR was achieved in four adult and four pediatric patients (57%), two adult patients fell under criteria of PR (14%), while two adult and two pediatric patients were non-responders (29%) (Table 3). The median cumulative dose in responders was 1,200 mg (range: 600–3,600), and in non-responders it was 1,200 mg (range: 600–1,200). The median time to overall response was 11 days (range: 2–27) (responders only). The corresponding Kaplan–Meier plot is presented in Figure 1A.

**Table 3:** Laboratory and clinical outcomes on 100 days and 1 year after the first dose of eculizumab.

Patient N	Age	Diagnosis	Concomitant condition	TA-TMA onset			100 days after eculizumab			Response (complete, partial, no response) 100 days	alive/ dead, at 100 days after Ecu	1 year after eculizumab			Alive/ dead
				Hemoglobin, g/l	PLT, 10^9^/l	GFR, ml/min	Hemoglobin, g/l	PLT, 10^9^/l	GFR, ml/min/1.73m^2^			Hemoglobin, g/l	PLT, 10^9^/l	GFR, ml/min/1.73m^2^	
1	6	Disceratosis congenita	Severe chronic GVHD, viral and bacterial pneumonia	53	8	19	-	-	-	No	Dead	-	-	-	Dead TA-TMA
2	9	ALL	Acute GVHD, sepsis	59	3	77	-	-	-	No	Dead	-	-	-	Dead, Relapse
3	7	ALL	Acute GVHD, sepsis	70	7	115	112	580	168	Yes	Alive	119	466	174	Alive
4	7	SDS	Sepsis	61	8	155	133	202	131	Yes	Alive	132	243	149	Alive
5	14	T-ALL	Cystitis, pericarditis	58	13	86	103	100	133	Yes	Alive				Dead, Relapse
6	1	Osteopetrosis	Acute GVHD	45	17	174	117	317	190	Yes	Alive	129	419	210	Alive
7	46	AML	Sepsis, CRS	65	0	17	122	34	94	Yes	Alive	161	261	97	Alive
8	61	PMF	Sepsis	64	2	22	-	-	-	No	Dead	-	-	-	Dead, sepsis, TA-TMA
9	35	AML	Overlap_GVHD, sepsis, HHV6 reactivation^1^	54	6	30	-	-	-	No	Dead	-	-	-	Dead, GVHD, sepsis
10	53	MDS	Sepsis	68	0	22	-	-	-	Partial	Dead	-	-	-	Dead, sepsis, VOD
11	34	MDS from disceratosis congenita	Sepsis	38	5	80	67	35	111	Partial	Alive	-	-	-	Dead, second tumor
12	35	HL	Acute GVHD	47	6	42	85	34	60	Yes	Alive	112	341	55	Alive
13	56	AML	CRS	85	4	40	90	40	97	Yes	Alive	125	137	99	Alive
14	18	MDS from Fanconi anemia	Cystitis, overlap GVHD, HHV6 reactivation	79	8	131	87	47	133	Yes	Alive	129	182	124	Alive

1 HHV6 - human herpes virus type 6.

After eculizumab treatment, 89% of patients experienced a 25% reduction in LDH levels, and 73% achieved nearly normal LDH levels (≤1.5× the upper reference limit). The median time of LDH response was 11 days (range: 2–27) and 22 days (range: 2–170), respectively. Additionally, 74% of patients showed improvement in platelet level, either a 50% increase or stabilization at ≥20 × 10^9^/L, while 56% reached platelet counts of ≥50 × 10^9^/L in 75 days (range: 5–384). The median time for platelet improvement was 21 days (range: 1–104) and 75 days (range: 5–384), respectively. Finally, 59% had an increase in hemoglobin of at least 1.0 g/dL without recent RBC transfusions with a median time of response of 22 days (range: 10–106) (Figures 1B–F).

Out of the 14 patients, 11 recovered to normal GFR/baseline GFR values after treatment, with a median time of 11 days (range: 2–26). Seven of 14 patients still had nephrotic-range proteinuria 30 days after the last eculizumab infusion. No patients were left dependent on dialysis.

TA-TMA-associated arterial hypertension was resolved in all four patients with a median time of 9 days (range: 1–27).

CNS involvement manifestations of TA-TMA resolved in three of six patients within 1–7 days from the first eculizumab infusion.

**Figure 2:**
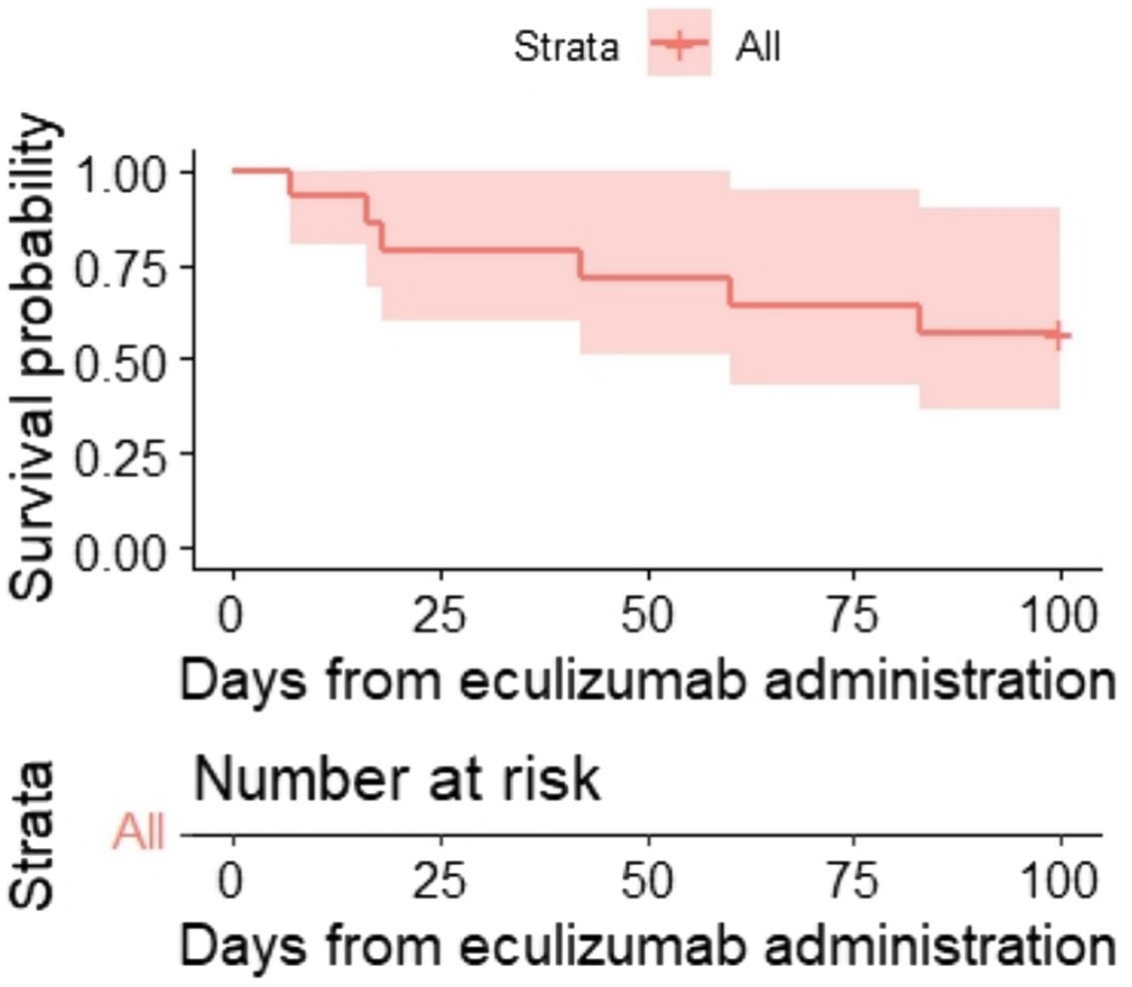
Kaplan–Meier plot for 100-day OS.

**Figure 3:**
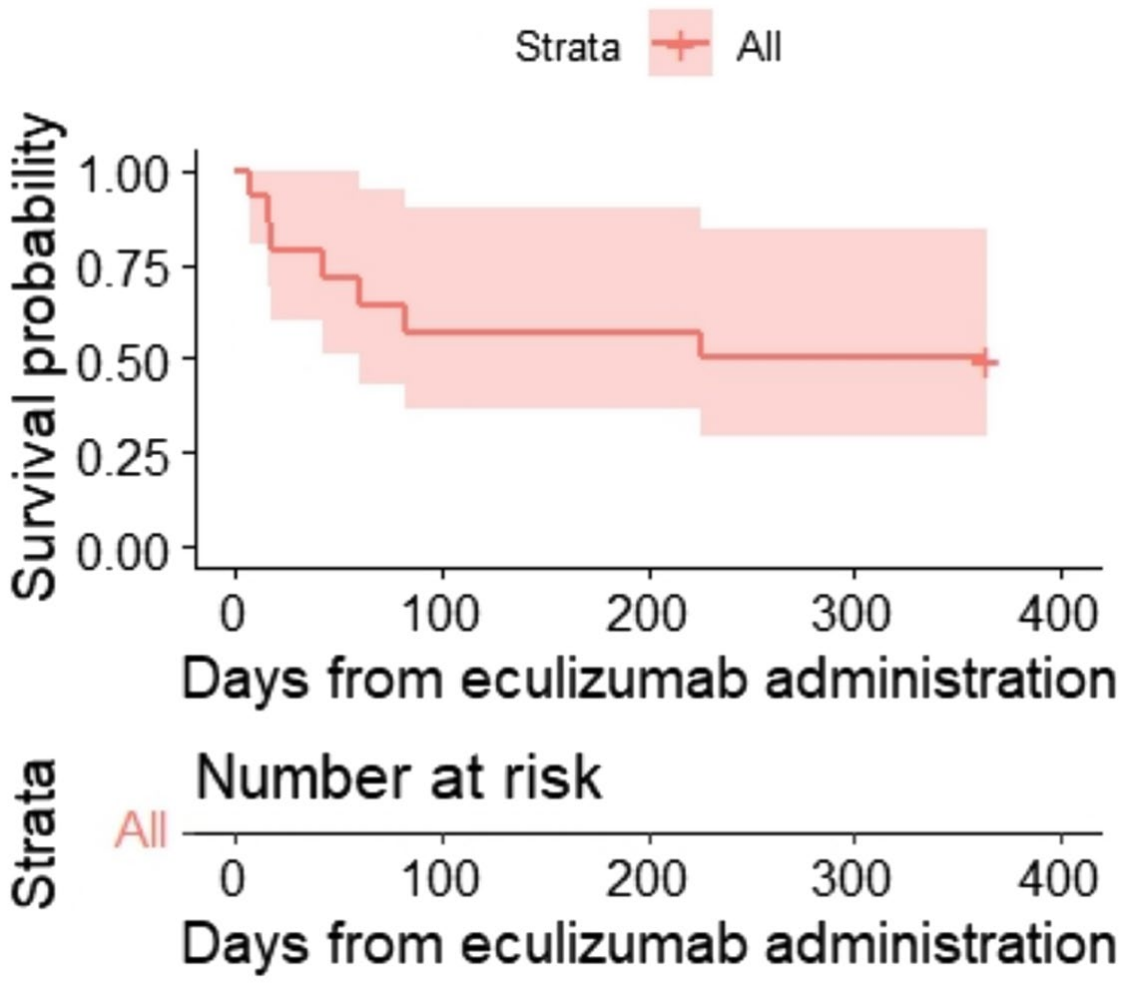
Kaplan–Meier plot for 1-year OS.

One hundred-day OS and 1-year OS were 57% (95% CI, 36–90) and 50% (95% CI, 30–84) (Figures 2, 3), respectively. One hundred-day OS for adults was 63% vs. 50% for pediatric patients, and 1 year OS was 50% vs. 50%, respectively. The *p*-value was not calculated due to small groups.

A total of eight subjects died, and two of them had active laboratory signs of TMA at death. Other causes of death were relapse (*n* = 2), infection (*n* = 2), GVHD (*n* = 1), and veno-occlusive disease (VOD) (*n* = 1). The median time from the last eculizumab dose to death was 52 (range: 10–546) days.

## Discussion

The complement system is a complex protein cascade involved in the early non-specific immune response. The vascular endothelium is constantly exposed to plasma complement, which could produce a strong proinflammatory signal if activated on the vascular wall (14). According to the “double-activation theory,” endotheliopathy can be induced by the activated complement system in critical conditions. This leads to two distinct molecular dysfunctions: activation of the inflammatory pathway through the release of cytokines such as interleukin 6 and tumor necrosis factor-*α*, and activation of the microthrombotic pathway through the exocytosis of hemostatic factors, e.g., large von Willebrand factor multimers and FVIII (15). Endothelial cell activation with accompanying vascular inflammatory changes is considered a common underlying mechanism in a number of different post-transplant complications (16–18). Conditioning regimen, graft–versus-host disease prophylaxis and GVHD itself, infections, and other allo-HSCT complications could serve as both solitary or cumulative triggers for endothelial damage. Over recent decades, the role of dysregulated complement activation in endothelial damage has been extensively demonstrated in certain forms of thrombotic microangiopathy (19–21).

The role of C5-derived peptide as a potent promoter of granulocyte and monocyte adhesion to endothelium and granulocyte autoaggregation was shown in 1979 by Craddock et al. (22). Mii et al. (23), and Laskin et al. (24) have demonstrated glomerular deposition of C4d as evidence of complement activation in TA-TMA proven by renal biopsy or autopsy. Further studies showed increased plasma levels of soluble terminal complement complex (sC5b-9) in patients with TA-TMA (25). Recently, Jodele et al. proposed a model of “interferon-complement loop,” which may maintain endothelial injury and thrombotic microangiopathy (26).

The use of eculizumab, a C5 complement inhibitor, for the treatment of transplant-associated thrombotic microangiopathy has shown encouraging results. The data from our study indicate that eculizumab treatment resulted in a complete response (CR) in 57% of patients. These results correlate closely with the data reported by Jodele et al., who demonstrated eculizumab-induced CR in approximately 60% of pediatric patients with TA-TMA, and with a systematic review by Zhang et al., showing an overall response of 71% in the treated patients (12, 27). These outcomes are significant, given the high mortality and morbidity associated with TA-TMA. However, the reported numbers of patients with full response vary in different studies, ranging from 32 to 57% (12, 27).

Despite the promising results, our study has several limitations. The small sample size and single-center design limit the generalization of our findings. Additionally, the retrospective nature of the study introduces potential biases, including selection and recall bias. Our study did not have complement activation control, and no study of polymorphisms associated with endothelial damage was performed. Moreover, the duration of eculizumab therapy was limited by the availability of the drug.

However, we described clinical outcomes in both children and adults, whereas most previous findings were primarily focused on the pediatric cohort. The usage of eculizumab for the treatment of TA-TMA in adults was described mostly in single clinical cases. There were no documented significant differences between adult and pediatric patients in our small cohort.

The effect of complement blockage in our group of patients with severe concomitant conditions treated with a reduced dosage regimen of eculizumab administration was still promising. According to our observations, the improvement in renal function was observed, i.e., reduced proteinuria, normalization of thrombocytopenia, and LDH levels among responders in both children and adults. However, concomitant conditions such as cytokine release syndrome, GVHD, and sepsis may have influenced the dynamics of TA-TMA manifestations. Further studies are required to define more decent criteria for partial and complete responses.

The small size and heterogeneity of our cohort still prevent any correlations between pre-transplant characteristics, laboratory findings, and clinical response. Nevertheless, we observed better renal outcomes in patients without pre-transplant proteinuria. Similarly to the data published by Jodele et al., we observed unfavorable results when eculizumab therapy was started at the terminal stages of TA-TMA with advanced organ failure and multiple concomitant conditions (27, 28).

The arrangement of timing and duration for the complement control therapy is one of the critical aspects of TA-TMA management. Our group practiced a relatively early therapy start, with the initiation of eculizumab therapy after 5 days, at a range of 0 to 30 days. Recent studies suggest that prompt initiation of eculizumab and a more aggressive dosing strategy might be beneficial for achieving better outcomes since early intervention can prevent the progression of endothelial injury and organ dysfunction. A tailored approach to dosing, potentially guided by biomarkers of complement activation and endothelial injury, could enhance therapeutic efficacy and minimize unnecessary treatment (8, 12, 28–31).

## Conclusion

Our study supports the use of eculizumab as a viable and effective treatment option for patients with TA-TMA following allo-HSCT. However, half of the patients still exhibited suboptimal responses; thus, further investigations of the correlation between TA-TMA manifestation response and survival are needed.

## Data Availability

The original contributions presented in the study are included in the article/Supplementary material, further inquiries can be directed to the corresponding author.
