# Supplementary material for: Time course of organ and hematological response to complement blockage in transplant-associated thrombotic microangiopathy after allogeneic hematopoietic stem cell transplantation
Source: Front Med (Lausanne). 2025 Mar 27;12:1551066. doi: 10.3389/fmed.2025.1551066 (PMC11983515; doi:10.3389/fmed.2025.1551066)
Supplement: Supplementary file 1 [file Table_1.DOCX]

**Supplementary materials**

Table 4. TA-TMA criteria

| **Parameter** | **Cho et al. (2010)** | **TMA Harmonization Panel Consensus Recommended Diagnostic Criteria (2022)** |
| --- | --- | --- |
| Criteria | All features present at ≥2 time points | Must meet ≥4 of the following 7 criteria within 14 days at 2 consecutive time points |
| Anemia | X | Defined as one of the following: 1. Failure to achieve transfusion independence for pRBCs despite evidence of neutrophil engraftment 2. Hemoglobin decline from patient’s baseline by 1 g/dL 3. New onset of transfusion dependence |
| Thrombocytopenia | X | Defined as one of the following: 1. Failure to achieve platelet engraftment 2. Higher than expected platelet transfusion needs 3. Refractoriness to platelet transfusions 4. 50% reduction or greater in baseline platelet count after full platelet engraftment |
| Elevated LDH | X | >ULN for age |
| Schistocytes | X (≥2 per HPF) | Present |
| Decreased haptoglobin | X |  |
| Negative Coombs test | X |  |
| Normal coagulation studies | X |  |
| sC5b-9 >ULN |  | X* |
| Proteinuria |  | ≥1 mg/mg rUPCR |
| Hypertension |  | >99th percentile for age (<18 yr), or systolic BP ≥140 mmHg or diastolic BP ≥90 mmHg (≥18 yr) |
| Biopsy |  | Or biopsy-proven disease (kidney or GI)** |

*: Due to limited availability sC5b-9 monitoring was not performed

** No biopsy was performed
